# Towards Faster Fetal 3D CINE Cardiac MRI Reconstruction Using Region‐Optimized Virtual Coils (ROVir)

**DOI:** 10.1002/mrm.70590

**Published:** 2026-09-22

**Authors:** Marjolein Piek, Johannes Töger, Einar Heiberg, Katrin Fricke, Erik Hedström, Anthony H. Aletras

**Affiliations:** ^1^ Clinical Physiology, Department of Clinical Sciences Lund Lund University Lund Sweden; ^2^ Department of Clinical Physiology Skåne University Hospital Lund Sweden; ^3^ Department of Biomedical Engineering Faculty of Engineering, Lund University Lund Sweden; ^4^ Pediatrics, Department of Clinical Sciences Lund Lund University Lund Sweden; ^5^ Pediatric Cardiology, Pediatric Heart Centre Skåne University Hospital Lund Sweden; ^6^ Diagnostic Radiology, Department of Clinical Sciences Lund Lund University Lund Sweden; ^7^ Department of Radiology Skåne University Hospital Lund Sweden; ^8^ School of Medicine, Laboratory of Computing, Medical Informatics and Biomedical ‐ Imaging Technologies, Aristotle University of Thessaloniki Thessaloniki Greece

**Keywords:** 3D, compressed sensing, fetal cardiac MRI, parallel imaging, radial, ROVir

## Abstract

**Purpose:**

To develop and evaluate, in a feasibility study, a computationally efficient image‐reconstruction pipeline for 3D balanced steady state free precession (bSSFP) CINE fetal cardiac MRI that reduces radial streaking artifacts, increases effective temporal resolution, and improves diagnostic image quality compared with previous work.

**Methods:**

Three‐dimensional bSSFP CINE data from 10 volunteers were used. Coil compression was performed using the region‐optimized virtual coils (ROVir) technique and compared with singular value decomposition (SVD) coil compression. For ROVir, the region of interest encompassed the fetal thorax, while the region of interference was positioned over the maternal subcutaneous fat to suppress unwanted signal contributions. Image reconstruction was performed for multiple temporal samplings. Two observers evaluated diagnostic image quality using a visual‐analog scale, and results were analyzed using visual grading characteristics (VGC). Computational efficiency was evaluated using reconstruction runtime and memory usage.

**Results:**

Average 3D CINE acquisition time was 5 min, with a reconstruction time of approximately 5 h for both SVD (8channels and 8 phases) and ROVir (3 channels and 16 phases), with approximately equal memory usage. ROVir enabled higher temporal resolution by reducing the channels required. VGC analysis showed a preference for ROVir across all diagnostic criteria (AUC > 0.5), including edge sharpness, perceived image contrast, artifact suppression, papillary muscles, great vessels, and extracardiac diagnostic image quality, with comparable median scores across temporal samplings.

**Conclusion:**

ROVir coil compression improved diagnostic quality and reduced reconstruction time. By reducing memory demands, reconstruction with a higher number of cardiac phases was also possible.

## 1 Introduction

Congenital heart defects (CHD) occur in approximately 1%–2% of all live‐births in western society and are the most common defect present at birth [1]. Prenatal diagnosis improves postnatal outcomes by enabling timely intervention and prenatal planning [2, 3, 4, 5]. While ultrasound is and will remain to be the first‐line standard of care for prenatal diagnosis, fetal cardiovascular magnetic resonance imaging (MRI) offers complementary diagnostic information. In fetal cardiac imaging, MRI is particularly useful in later gestation when acoustic windows may be limited [6]. Additional indications include high maternal body mass index and assessment of pulmonary lymphangiectasia in relation to CHD. Nevertheless, fetal cardiac MRI remains challenging due to the small size of the fetal heart, high heart rates, fetal motion, and the unavailability of reliable ECG gating.

To date, fetal cardiac MRI has mainly relied on two‐dimensional (2D) imaging. The development of fetal cardiac CINE MRI has been enabled by advances in cardiac gating, motion correction, and image reconstruction techniques, which have addressed some of the challenges associated with fetal motion and the absence of reliable ECG gating [7, 8]. Yet, due to the complex anatomy of CHD and the limited in‐plane resolution of 2D acquisitions, accurate diagnosis remains difficult. Three‐dimensional (3D) reconstruction has been proposed to overcome these limitations [9, 10, 11, 12]. Black‐blood slice‐to‐volume reconstruction techniques have shown promise in visualizing vascular structures, but their lower temporal resolution restricts their application to dynamic cardiac imaging [13]. To improve temporal fidelity, balanced steady‐state free precession (bSSFP) slice‐to‐volume reconstruction methods were introduced, enabling dynamic visualization of fetal cardiac motion [14].

Building upon prior developments in fetal cardiac CINE acquisition and reconstruction, the first true 3D bSSFP CINE fetal cardiac MRI acquisition enabled direct capture of volumetric cardiac motion and thus cardiac volume and function, rather than relying on slice‐to‐volume reconstruction from 2D slices [15]. This acquisition has shown potential for improving the diagnosis of congenital heart defects, but its current implementation is affected by motion artifacts, suboptimal diagnostic quality, inherently low blood‐to‐myocardium contrast, and considerable computational demands. Computational demands are substantial due to the large maternal field of view (FOV) and the number of receiver coils, often necessitating trade‐offs between reconstruction complexity, temporal resolution, and the number of cardiac phases.

The current feasibility study focuses on addressing computational limitations with the overall goal of moving the 3D bSSFP CINE fetal cardiac MRI method closer to clinical feasibility. Diagnostic image quality is further affected by radial streaking artifacts arising from strong maternal subcutaneous fat signal. Current reconstruction methods rely on singular value decomposition (SVD) for coil compression, which prioritizes the strongest signals across the entire image, which mainly come from the maternal subcutaneous fat signal in fetal MRI instead of signals coming from the fetal heart.

Therefore, the aims of this study were to evaluate whether the use of Region‐Optimized Virtual Coils (ROVir) [16] method in 3D bSSFP CINE fetal cardiac MRI could (i) reduce computational demands, (ii) decrease radial streaking artifacts, and (iii) enable reconstruction with a higher number of cardiac phases, thereby supporting further progress towards clinical implementation. To this end, ROVir, a recently introduced coil compression method, was implemented and compared with standard SVD reconstruction. The goal was to emphasize signals within a predefined region of interest, namely the fetal heart, and attenuate the surrounding maternal subcutaneous fat. Since ROVir prioritizes signals from the region of interest, it was hypothesized that ROVir could reduce the number of cardiac coils required for reconstruction, permitting higher temporal resolution reconstructions with similar hardware.

## 2 Methods

### 2.1 Study Design

This study was performed at Skåne University Hospital, Lund, Sweden, in accordance with the Declaration of Helsinki and approved by the Regional Ethics Review Board in Lund, Sweden (Dnr 2013/551), and is reported according to the STROBE guidelines [17]. All pregnant women consented in writing prior to participation.

Since 2019, 68 pregnant women in gestational weeks 32–40 have been scanned using the 3D bSSFP CINE fetal cardiac sequence. The cohort included healthy volunteers and women carrying a fetus with suspected congenital heart defects (CHD). Exclusion criteria were fertility treatment for the healthy volunteers and contraindications for fetal cardiac MRI for all.

For evaluation of the proposed methods, a subset of 10 cases (median gestational age 37 weeks) with minimal fetal motion was selected (Table 1). Fetal bulk motion was assessed by visual inspection of low‐resolution temporal reconstructions generated without cardiac binning, as previously described [15]. These reconstructions were used to identify periods of fetal motion and to determine whether sufficient motion‐free data were available for reconstruction. Only datasets exhibiting minimal fetal bulk motion throughout the acquisition were included in the present analysis.

**TABLE 1:** Subject characteristics for the 10 included fetuses.

Subject characteristics			Imaging parameters						
Case	GA	Path.	HR (bpm)	FA	Res (mm^3^)	FOV (mm)	Read‐outs	TA (mm:ss)	Coil elements
1	32 + 2	Borderline LV, suspected CoA	113	46°	1.9	720	65 500	3:52	34
2	37 + 4	D‐TGA‐IVS	148	40°	1.7	640	100 000	5:40	34
3	36 + 4	Borderline LV, suspected CoA	154	45°	1.9	720	100 000	5:29	34
4	37 + 5	—	133	42°	1.9	720	100 000	5:29	50
5	36 + 3	D‐TGA‐IVS	141	46°	1.7	640	100 000	5:40	30
6	36 + 6	Small LV, arch hypoplasia, suspected CoA	125	45°	1.9	720	100 000	5:23	30
7	36 + 1	—	127	45°	1.9	720	70 000	3:52	30
8	37 + 5	—	122	48°	1.9	720	65 500	3:37	34
9	34 + 6	—	121	46°	1.9	720	65 500	3:37	30
10	37 + 4	DORV	138	40°	1.9	720	65 500	3:37	34

Abbreviations: bpm, beats per minute; CoA, coarctation of the aorta; DORV, double outlet right ventricle; D‐TGA, D‐transposition of the great arteries; FA, flip angle; FOV, field of view; GA, gestational age; HR, heart rate; IVS, intact ventricular septum; LV, left ventricle; Path., pathologies; res, resolution; TA, acquisition time.

Aside from healthy volunteers, the cohort included fetuses with borderline left ventricle, D‐transposition of the great arteries with intact ventricular septum, small left ventricle, aortic arch hypoplasia, suspected coarctation of the aorta, and double outlet right ventricle. Recorded fetal heart rates were 130 beats per minute (range: 113–154).

Including a 19‐s localizer, the 3D CINE acquisition time was 5 min per subject (range: 3.9–6 min), corresponding to 65 500–100 000 radial spokes. The acquired voxel size was 1.7–1.9 mm^3^ with a FOV of 640–720 mm. Typically, 34 (range: 30–50) of the 50 available coil elements were used for data acquisition.

### 2.2 Magnetic Resonance Imaging

A 1.5T scanner, with an anterior 18‐channel phased array and a 32‐channel spine phased array (MAGNETOM Aera, Siemens Healthineers, Forchheim, Germany), was used for fetal cardiac MRI. Fetal cardiac gating was performed using the smart‐sync device (Northh medical GmbH, Hamburg, Germany) [18]. The women were positioned in the left lateral decubitus position during the entire examination to avoid compression of the inferior vena cava.

A 3D slab‐selective radial bSSFP research pulse sequence with a double golden‐angle radial k‐space trajectory [19] was used for data acquisition in pregnant women as described in previous work [15]. In brief, the 3D imaging volume covered the fetal thorax and a typical acquisition took 3.6–5.8 min, had a TR of 3.3–3.5 ms, an isotropic voxel size of 1.6–1.9 mm^3^ with a FOV of 640–720 mm, used a 40°–48° flip angle and a bandwidth of 1085 Hz/pixel. Examinations were performed at normal operating mode, with specific absorption rate constrained to 2 W/kg.

### 2.3 Image Reconstruction

Figure 1 shows the 3D CINE image reconstruction pipeline. The Berkeley Advanced Reconstruction Toolbox (BART v.0.9.00) [20] and Matlab R2024a (MathWorks, Natick, MA, USA) were used for image reconstruction. All image analysis was done using Segment v4.1.0.0 R15011 (Medviso AB, Lund, Sweden) and Matlab R2022a [21].

**FIGURE 1:**
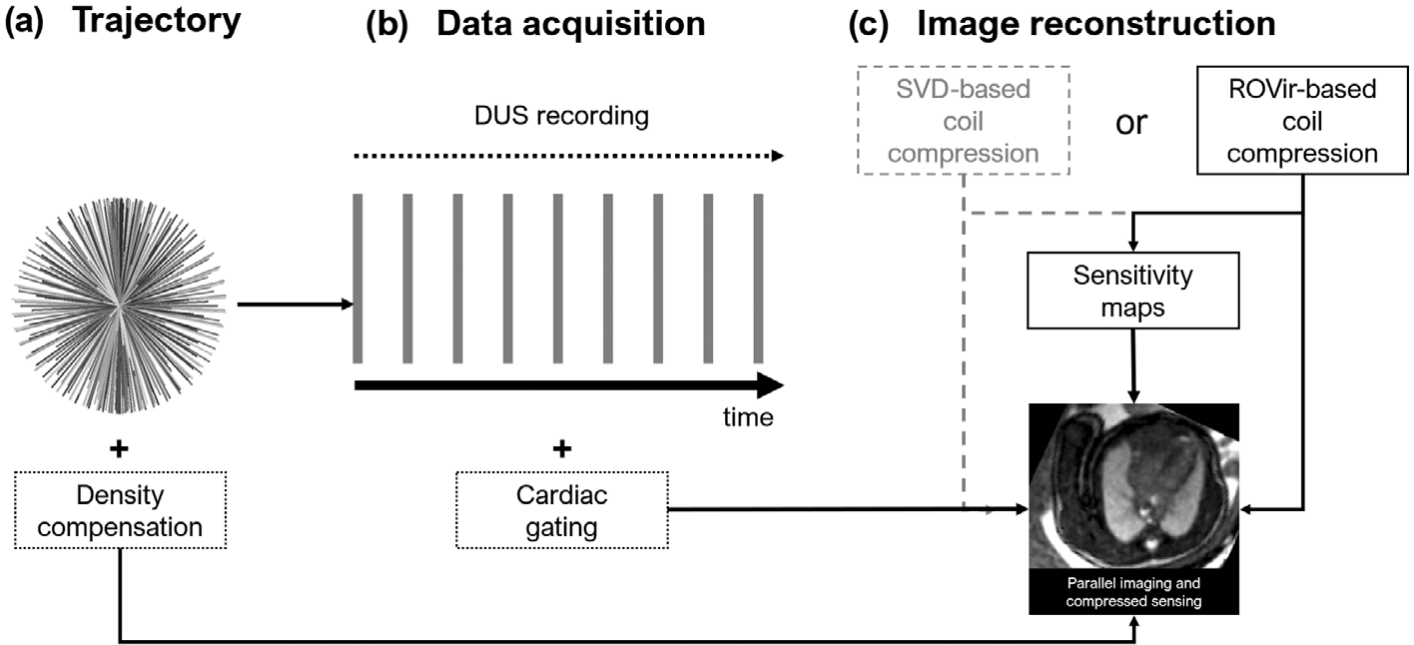
Flowchart of the fetal image acquisition and reconstruction pipeline with ROVir coil compression. (a) The 3D radial double golden‐angle (“Kooshball”) trajectory used to acquire k‐space data. (b) Doppler ultrasound (DUS) signals were acquired simultaneously with k‐space data during the cardiac MRI acquisition. (c) Image reconstruction steps included coil compression based on SVD or ROVir, estimation of virtual coil sensitivity maps and parallel imaging and compressed sensing.

Coil compression on all channels, $N_{c}$, was performed using two methods, SVD and ROVir. For SVD, coil exclusion was performed manually to remove channels dominated by noise or signal from outside the region of interest, as previously described [15]. In contrast, the ROVir pre‐processing included an automatic exclusion of coils affected by radial streaking artifacts [22].

#### 2.3.1 ROVir Coil Compression

The 3D spatial regions for the region of interest and the region of interference were manually defined by an experienced observer (M.P.; biomedical engineer, 7 years' experience in fetal cardiac MRI) using the software Segment 3DPrint version 4.1.1.0 (Medviso AB, Lund, Sweden). The left panel in Figure 2 shows how the region of interest was defined to cover the fetal thorax, and the region of interference was placed over the brightest areas of maternal subcutaneous fat, avoiding overlapping with the region of interest. Based on these two regions, the inter‐coil signal correlation matrices were computed and generalized eigenvalue decomposition was performed to obtain the generalized eigenvalues and eigenvectors. After sorting the eigenvalues and eigenvectors, the virtual k‐space data were computed. In this way, the first channel preserved most of the signal in the volume of interest, while the last channel preserved most of the signal in the region of interference. Reconstructions were performed using 3 and 8 compressed channels, representing aggressive and moderate coil compression, respectively. These values were selected a priori and used throughout the study to assess the performance of SVD and ROVir at different compression levels.

**FIGURE 2:**
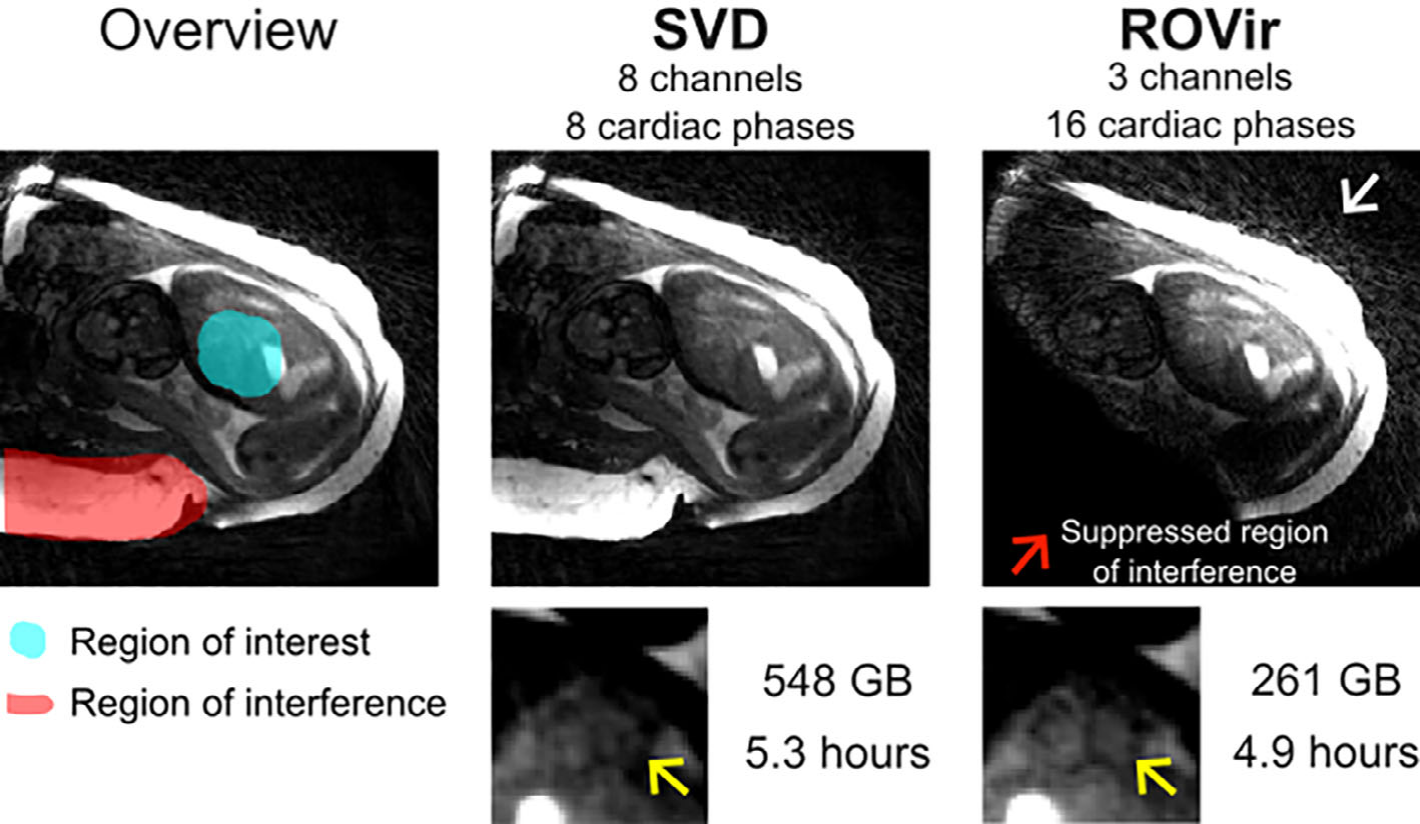
Illustration of the ROVir approach. The left panel shows the interference region over maternal subcutaneous fat (red) and the region of interest over the fetal heart (blue). The middle panel shows an SVD‐based reconstruction using 8 virtual coils and 8 cardiac phases. The right panel shows an ROVir‐based reconstruction using 3 virtual coils and 16 cardiac phases, where the number of coils is reduced while maintaining signal within the region of interest. The red arrow indicates suppression of the interference region. The observed increase in radial streaking (white arrow) in the ROVir reconstruction occurs outside the region of interest and does not affect the depiction of the fetal heart. Two zoomed images are provided to facilitate visualization of the fetal heart, and show short‐axis views with a yellow arrow indicating the left ventricle.

#### 2.3.2 Parallel Imaging and Compressed Sensing

After coil compression, sensitivity maps were calculated using ESPiRIT (BART command: ecalib) [23] and the radial spokes were binned to their corresponding cardiac phase based on the Doppler ultrasound triggers. The Nyquist criterion dictates that $\left(4\pi r^{2}\right)/2$ spokes should be binned to each cardiac phase, where 2r is the number of samples per radial spoke. The Nyquist criterion was evaluated at the edge of a spherical FOV with twice the prescribed radius. The acceleration rate (*R*) was then calculated by dividing the Nyquist limit by the number of binned spokes per cardiac phase.

A parallel imaging and compressed sensing algorithm was used to solve the nonlinear minimization problem iteratively with total variation over the temporal domain. For the ROVir reconstruction, 8 and 16 cardiac phases were reconstructed for all datasets. All reconstructions used a temporal regularization parameter $\lambda_{t}=0.001$ and 50 iterations. For the SVD reconstruction, the same datasets and phases were reconstructed, in a similar way to earlier work [15]. All reconstructions were performed on a server using 16 threads on an 18‐core Intel Xeon Gold 5520R CPU (2.2 GHz base, 4.0 GHz turbo) with 1.5 TB random access memory (RAM) or on a 32‐core AMD Ryzen Threadripper PRO 9975WX CPU (4.0 GHz base, 5.4 GHz turbo) with 1 TB RAM. Reconstruction time and peak RAM use were logged on the 1.5 TB server.

### 2.4 Image Evaluation

Standard cardiac views were obtained by multiplanar reformatting in Segment prior to image evaluation. For functional interpretation, images were reconstructed to their original isotropic pixel size. Visual grading was performed by two observers, observer 1 (E.H.; pediatric radiologist, 25 years' experience in cardiac MRI and 11 years' in fetal cardiac MRI) and observer 2 (K.F.; pediatric and fetal cardiologist, 11 years' experience in fetal echocardiography and 6 years' in fetal cardiac MRI). A total of 40 image datasets were evaluated, corresponding to reconstructions from 10 subjects using different reconstruction methods, numbers of compressed channels, and numbers of reconstructed cardiac phases. The observers were blinded to the reconstruction method and subject identity. The datasets were presented individually in randomized order. For each dataset, the observer evaluated an overview image together with transverse and sagittal CINE images. To assess the diagnostic impact, observers rated key image attributes relevant to fetal cardiac evaluation (e.g., edge sharpness, artifact level, perceived image contrast) using a 7 cm visual analog scale, where 0 cm indicated the poorest and 7 cm the best perceived diagnostic image quality [24]. The visual grading process produces ordinal ratings, as they represent categories of diagnostic image quality and may not be treated as numerical values. Using SVD as reference (*x*‐axis) and ROVir as test (*y*‐axis), VGC Analyzer (version 1.0, release 3) computed VGC curves by sweeping a threshold along the 0–7 cm scale and plotting, at each threshold, the cumulative proportion of images with scores ≥ threshold for SVD versus ROVir [25, 26, 27, 28, 29]. The area under the VGC curve (AUC) and corresponding confidence intervals (CI) are reported together with *p* values from the VGC random‐reader analysis. These *p* values are presented descriptively and interpreted in conjunction with the AUC and CI, without applying a predefined significance threshold, in accordance with recommended practice for VGC analysis [28]. Interobserver consistency for each image quality criterion was assessed using a two‐way mixed‐effects intraclass correlation coefficient for consistency of average measurements (ICC(3,*k*), where *k* = 2 observers). ICC values were interpreted as poor (< 0.50), moderate (0.50–0.75), good (0.75–0.90), and excellent (> 0.90), according to Koo and Li [30]. Statistical analyses were performed using RStudio (version 2026.07.1 + 147; Posit Software, PBC, Boston, MA, USA) [31].

#### 2.4.1 Computational Efficiency

Computational efficiency was evaluated in terms of reconstruction runtime and memory usage. Reconstruction times were recorded for all subjects and reconstruction configurations. Results were summarized across all subjects as mean ± standard deviation. RAM usage was recorded for a representative dataset reconstructed with the same parameter combinations.

## 3 Results

### 3.1 Reconstruction

For all 10 cases, reconstructions were generated with 8 and 16 cardiac phases using 3 and 8 compressed channels. Additional reconstructions with 20 cardiac phases (*N* = 4) are provided in Figure S1.

Figure 2 illustrates the effect of reducing the number of virtual channels using the ROVir approach compared with a standard SVD‐based reconstruction. Compression to 3 ROVir virtual channels, compared with 8 SVD channels, reduces signal contributions from outside the predefined region of interest. Signal from the interference region, dominated by maternal subcutaneous fat, is substantially suppressed in the ROVir reconstruction, while signal within the fetal thorax is preserved. Residual signal from other maternal subcutaneous fat regions remains visible, as these regions are not included in the interference definition and are therefore not explicitly suppressed. This reflects the trade‐off between interference suppression and preservation of signal within the region of interest, given the spatial overlap of coil sensitivity profiles. Figure S2 shows all reconstruction combinations for two representative cases.

Figure 3 shows multiplanar reformatted CINE images at end‐diastole for two representative cases. Across all temporal samplings for 3 compressed channels, the ROVir images show sharper myocardial borders and an improved perceived image contrast compared to the SVD images. Videos S1 and S2 show a decrease in flickering artifacts for the ROVir images.

**FIGURE 3:**
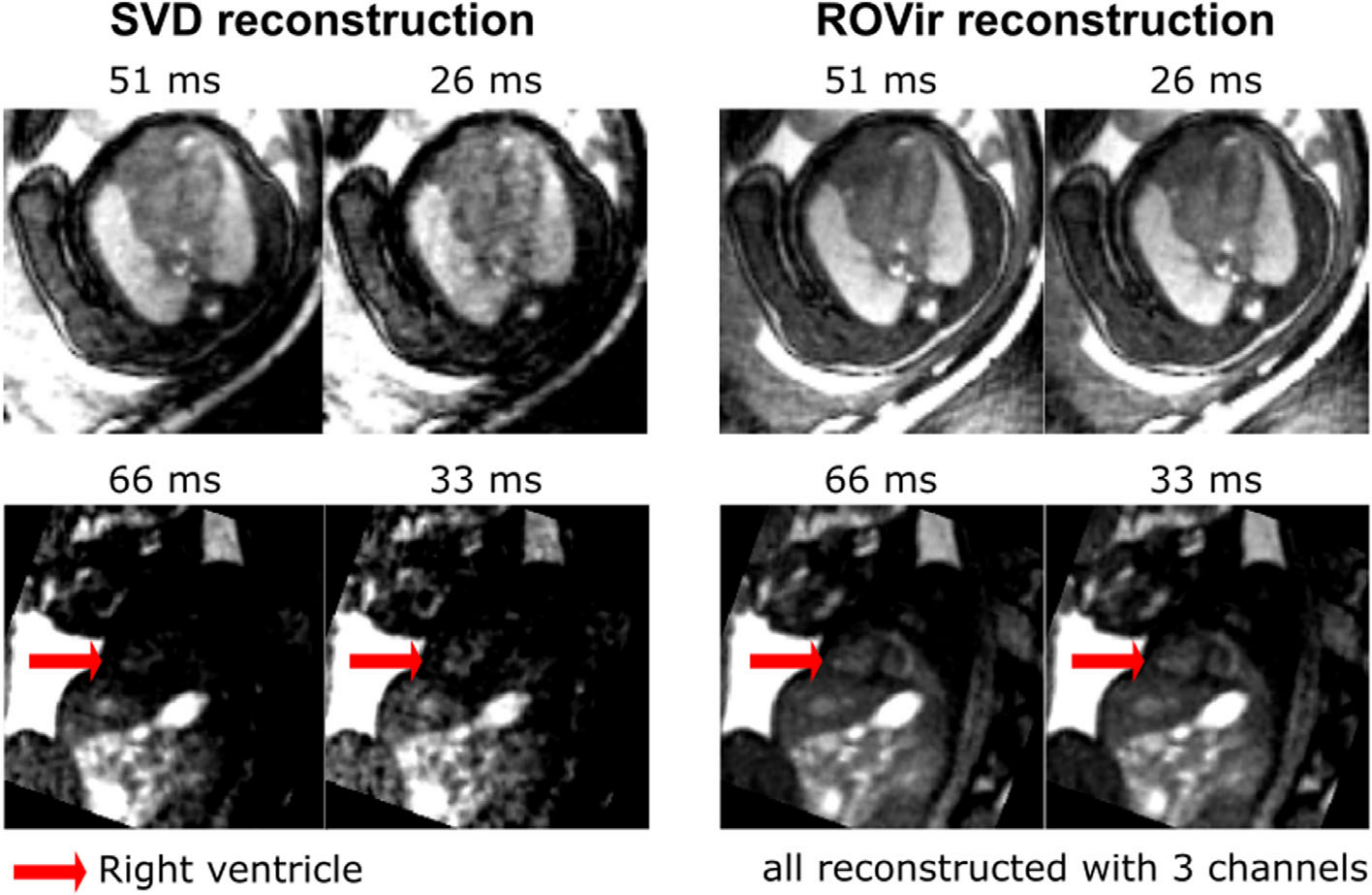
Multiplanar reformatted CINE images at end‐diastole in two cases: A case with D‐transposition of the great arteries (top panel) and a case with a borderline left ventricle (bottom panel; red arrows pointing at the right ventricle). The left panel shows the SVD reconstructions, and the right panel shows the corresponding ROVir reconstructions for two different temporal samplings. All were reconstructed using 3 compressed channels. Representative images are shown after inter‐slice interpolation applied for visualization purposes. Cardiac function for both cases, comparing the SVD‐ and ROVir reconstructions, are shown in Videos S1 and S2.

Figure S3 shows the region of interest and the region of interference for all cases and multiplanar reformatted CINE images comparing the SVD and ROVir reconstructions. Figure S4 shows the effect of the interference region position and size in one representative case.

Figure 4 shows computational demands and runtime for the parallel imaging and compressed sensing measured in one representative dataset. The acceleration factor varied from *R* = 15–42, for 8 and 16 cardiac phases, respectively. Reconstruction time was approximately 5 min per reconstructed cardiac phase and virtual channel.

**FIGURE 4:**
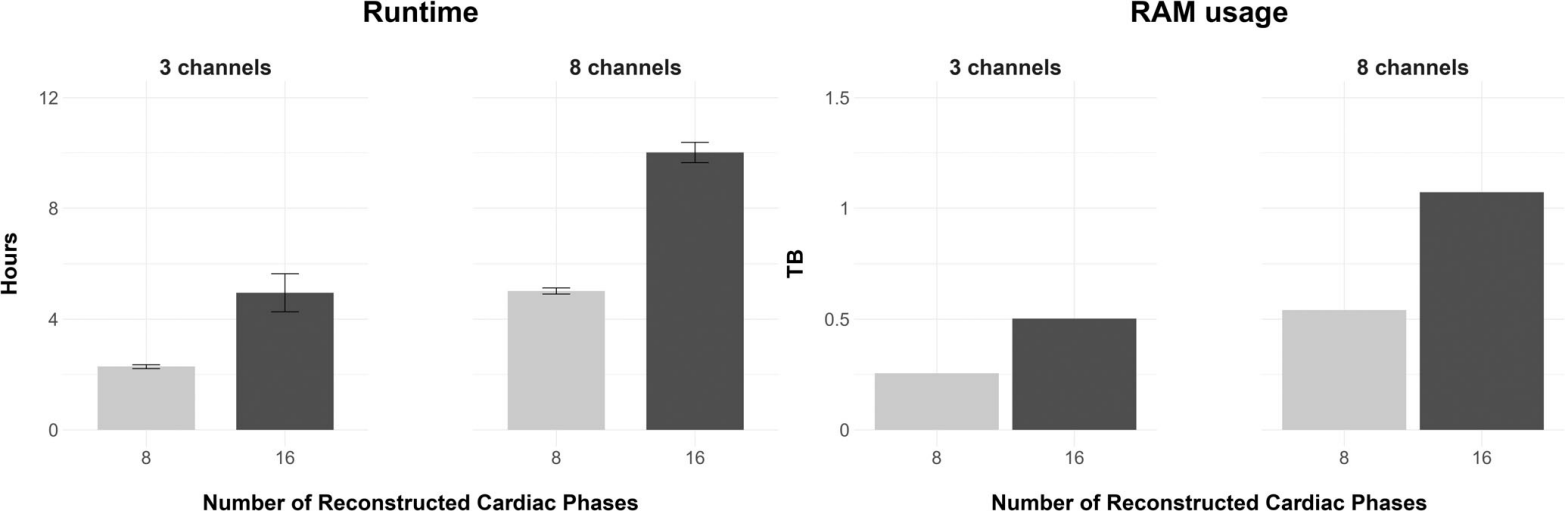
Mean reconstruction runtime across all subjects (mean ± SD) and RAM usage for a representative dataset for reconstructions using 3 and 8 compressed channels and 8 and 16 reconstructed cardiac phases. Reconstruction time increased from 2.3 ± 0.1 h (3 channels and 8 phases) to 10.0 ± 0.3 h (8 channels, 16 phases). RAM usage is shown descriptively as it was only tracked for a representative dataset. Notably, a 3‐channel, 16‐phase ROVir reconstruction required less computational time than the previously published [9] 8‐channel, 8‐phase SVD reconstruction.

### 3.2 Grading of Diagnostic Image Quality

Visual grading boxplots are shown in Figure 5. For both observers, the distribution of scores for ROVir shifted towards higher values, with higher median scores than for SVD. Statistical comparison of observer ratings was performed using VGC analysis. Individual criterion scores are reported in Table S1. Across all seven criteria, ROVir demonstrated numerically higher median scores than SVD for both observers. Interobserver consistency ranged from moderate to good, with ICC(3,*k*) values between 0.47 and 0.76. The lowest agreement was observed for absence of artifacts and extracardiac diagnostic image quality (both ICC = 0.47).

**FIGURE 5:**
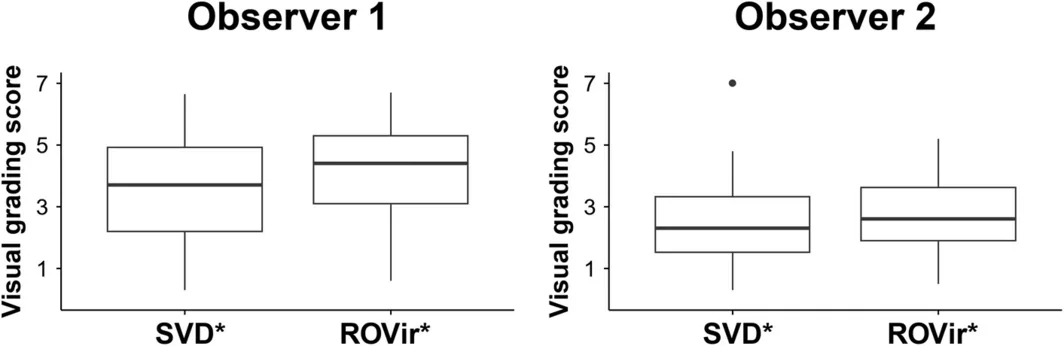
Box plots showing the visual grading scores for an experienced fetal cardiac MRI observer (25 years; left panel) and a less experienced fetal cardiac MRI observer (11 years; right panel). Each box represents the interquartile range, with the median as a horizontal line and dots representing outliers. The comparison is between the ROVir image reconstruction and the standard SVD image reconstruction. Both observers generally assigned similar or higher scores to the ROVir reconstructions. *For all number of channels and cardiac phases combined.

Figure 6 shows visual grading boxplots for the two different temporal samplings for the ROVir reconstructions. Median scores were comparable, and the interquartile ranges demonstrated consistent diagnostic image quality without evidence of degradation at higher temporal samplings. These results suggest that ROVir maintains diagnostic quality even when reconstructing at higher temporal samplings.

**FIGURE 6:**
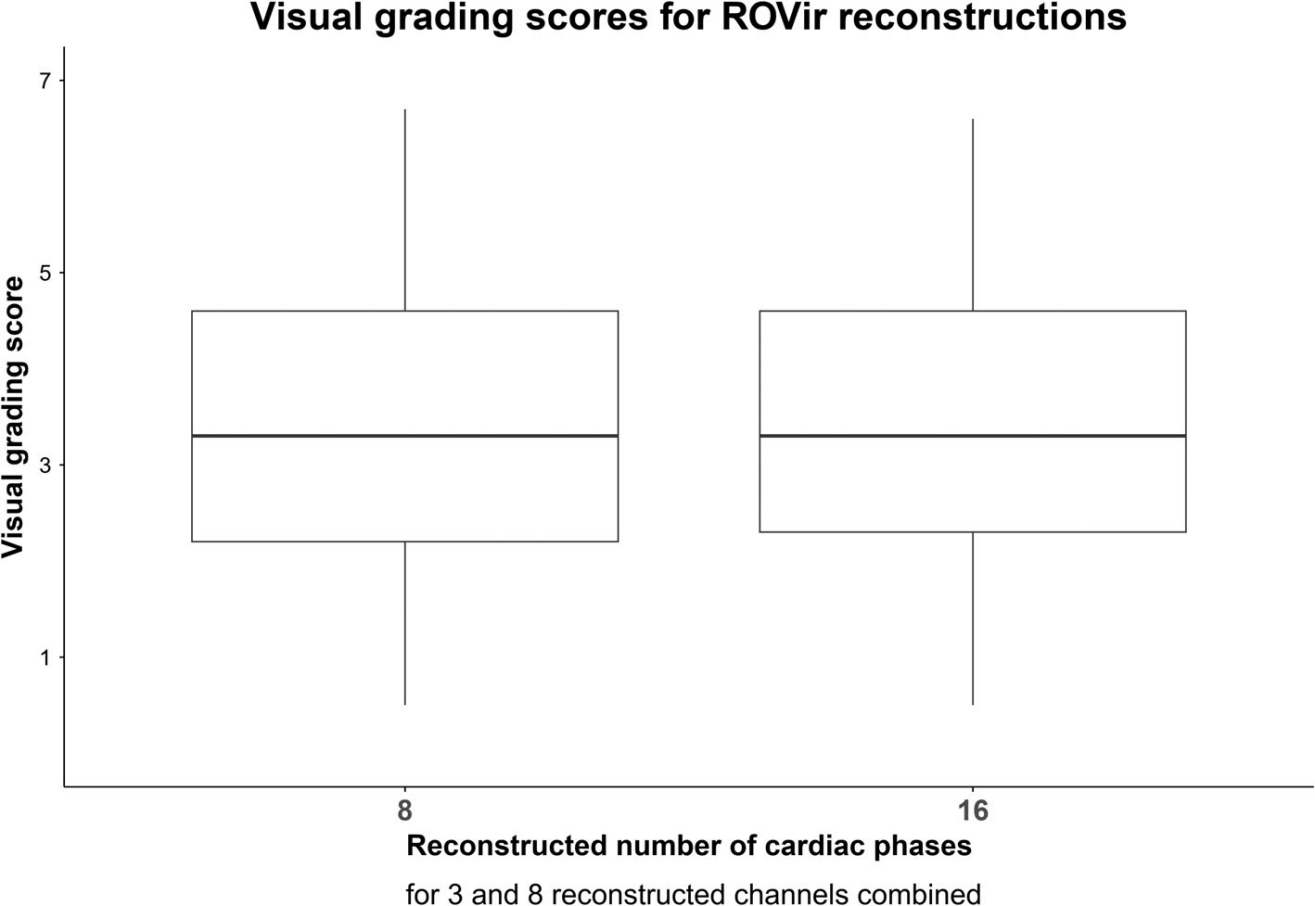
Box plots showing the combined visual grading scores of both observers for the ROVir reconstruction at two temporal samplings, corresponding to 8 and 16 reconstructed cardiac phases. Differences in the number of phases reflect changes in temporal resolution, which is between 25 and 66 ms, depending on heart rate. Results are shown for both observers combined. Note that they show largely overlapping score distributions. While the lowest temporal sampling had the highest median score, the overall differences between them were minor.

Figure 7 shows VGC curves for different diagnostic criteria for both observers combined. The area under the curve values ranged from 0.627 to 0.676 (95% CI: 0.515–0.769, *p* = 0–0.009). For Observer 1, the area under the curve values ranged from 0.6271 to 0.7118 across the evaluated factors (95% CI: 0.5108–0.7957; *p* = 0–0.0300). For Observer 2, the area under the curve values ranged from 0.5828 to 0.6793 (95% CI: 0.4770–0.7585, *p* = 0–0.1105). The area under the curve combined with the *p* values indicates that the ROVir reconstruction outperforms the SVD reconstruction for all criteria.

**FIGURE 7:**
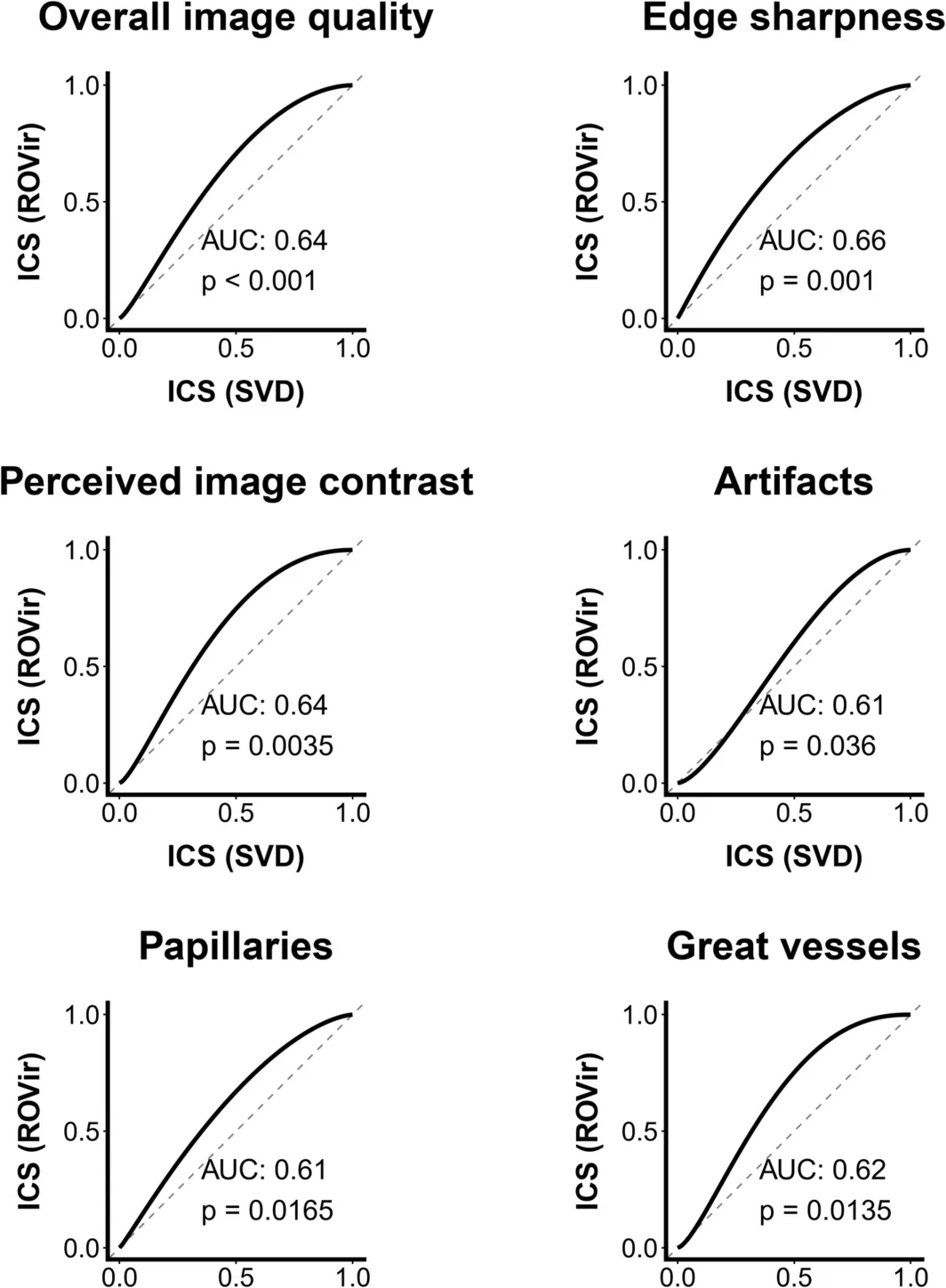
VGC curves for the different diagnostic quality criteria for both observers combined. Image quality was assessed using an ordinal image criteria score (ICS). The *x*‐axis shows the cumulative proportion of images with an ICS ≥ threshold for the SVD reconstruction (reference), and the *y*‐axis shows the corresponding proportion for the ROVir reconstruction (test). The area under the VGC curve (AUC) summarizes relative image quality. The dashed line represents the line of identity, corresponding to equal image quality between methods.

## 4 Discussion

A whole‐heart fetal 3D radial CINE MRI acquisition with a ROVir image reconstruction was implemented in this feasibility study to address three key limitations of the previously published SVD image reconstruction. The suggested approach (i) decreased the computational demands, (ii) increased overall diagnostic image quality, and (iii) enabled reconstruction with a higher number of cardiac phases, while maintaining at least equal diagnostic image quality. This proposed method thus moves fetal 3D CINE closer to clinical application.

To address image degradation caused by the severe undersampling, the ROVir coil compression method was used instead of the standard SVD compression. The ROVir method was implemented to enhance signal originating from the fetal heart while suppressing signal contributions from maternal subcutaneous fat, which frequently leak into the region of interest and degrade diagnostic image quality. This signal leaking was primarily assessed by the observers in the category “absence of artifacts”. Interobserver agreement was moderate to good across all evaluated image quality criteria. Lower agreement was observed for absence of artifacts and extracardiac diagnostic image quality, indicating that these criteria may be more dependent on individual observer interpretation than image contrast or sharpness. Further, visible artifacts were observed. However, their effect on diagnostic interpretation was not consistent and appeared to vary across observers with different levels of experience.

Additionally, a benefit of ROVir coil compression is its ability to lower the number of coils necessary for the parallel image reconstruction part. This reduction in coils translated to decreased RAM utilization and faster image reconstruction during the reconstruction process. The available RAM then allowed for additional cardiac phases to be reconstructed, thereby increasing the overall temporal resolution of the fetal 3D CINE volume. As fetal heart rates normally range between 120 and 160 bpm, temporal resolution needs to be high for accurate evaluation of cardiac function during the rapid fetal systolic and early diastolic motion. Increasing the temporal resolution from approximately 60 ms to 20–30 ms can help to better represent the fetal cardiac motion throughout the cardiac cycle [32].

Despite using ROVir coil compression, the 3D dataset remained large with an average isotropic dimension of 384 k‐space samples. This was due to the inherent oversampling factor of 2 in the radial readout and the size of the maternal abdomen and pelvis. In addition, the NUFFT implementation in BART performs gridding on an oversampled Cartesian grid with a factor of 2, which further increased the intermediate data size. High accuracy with a reduced gridding oversampling of a factor of 1.3 has been shown to be possible [33], and should be explored in future work. The BART toolbox used for the current work solved the optimization problem using an Alternating Direction Method of Multipliers (ADMM)‐based technique, which is known to be computationally demanding, especially in 3D reconstruction [34, 35, 36]. As future work, faster optimization strategies could be explored. Another approach could be to reduce the FOV, since it was acquired with a factor of 2 oversampling. Reducing the number of data samples per readout yields a cubic reduction in data size, which would have a significant impact on the reconstruction time, irrespective of the chosen optimization approach. Reducing the FOV is a potential solution, but must be balanced against the risk of residual aliasing if signal originating outside the reduced FOV is not sufficiently suppressed.

Having clinical applicability in mind, the reconstruction process should be automated. Using ROVir for reconstruction required manual annotation of the regions of interest and interference, limiting the utility of the method. Therefore, an automatic segmentation of the regions of interest and interference is required to make the method clinically widely applicable. This type of automatic segmentation could be implemented with a neural network, as previously used for gross fetal, placental and uterine segmentation [37, 38]. In theory, to automate the process, region of interest masking would involve localizing the fetal heart and using its position as the center point for a spherical mask. Approximating the region of interference is more challenging as it is not feasible to simply mask out all the high‐intensity maternal subcutaneous fat, since this envelops the region of interest of the fetal heart. However, as long as the interference region is not placed too close to the region of interest, ROVir appears relatively robust to variations in the size and placement of the interference region. The ROVir method relies on a linear combination of coils that have a strong sensitivity to the region of interest, but a much lower sensitivity to the region of interference. Therefore, only the coils with a good vantage point to the region of interest should be selected in the reconstruction. Automating this is critical for translating ROVir from a research tool to a clinically viable reconstruction method.

To evaluate cardiac anatomy and physiology, the reconstructed 3D CINE volumes were manually reformatted into clinically relevant 2D imaging planes. The 3D volumes were acquired in a maternal feet‐head orientation, and the first step was to reformat to standard transverse, sagittal, and coronal planes with respect to the fetal heart. From these orthogonal imaging planes, the clinically relevant imaging planes which depended on the cardiac abnormalities were then reformatted by an experienced observer. Being able to do this offline, when time was ample, was the main advantage of the 3D CINE sequence. In 2D cases, this would have to be done while the patient is in the scanner and time is an issue, both for maternal comfort and minimizing fetal bulk motion artifacts. Neural networks can potentially facilitate multiplanar reformatting by automatically localizing the fetal heart, estimating its orientation, and generating the clinically relevant imaging planes.

Due to the long scanning time, fetal motion and maternal respiration degraded the diagnostic quality of the 3D CINE fetal cardiac MRI images. Work on addressing these motion issues has been done on slice‐to‐volume reconstruction techniques in black‐blood imaging, where multiple 2D image stacks in different slice orientations are reconstructed to a 3D volume [9, 10]. Recent work proposed the automatic respiratory and bulk patient motion corrected (ACROBATIC) framework for correcting motion in 3D radial fetal MRI [8]. This framework increases edge sharpness by addressing both maternal respiratory motion and fetal bulk motion. Approaches for 3D CINE fetal cardiac MRI need yet to be developed. While recent progress on motion correction is promising, further advances will be essential for enabling robust, high‐quality diagnostic 3D fetal CINE imaging. Since ROVir is a coil compression technique and does not incorporate motion correction, the present study was limited to datasets with minimal fetal motion to enable evaluation of coil compression performance without the confounding effects of severe motion artifacts.

## 5 Conclusions

In conclusion, the feasibility of a ROVir‐enhanced 3D radial CINE MRI acquisition for improved fetal MR‐based cardiac imaging was demonstrated. The benefits of decreased computational demands, shorter acquisition time, and reduced radial streaking artifacts, coupled with reduced memory requirements enabling reconstruction of a higher number of cardiac phases that is key to measure cardiac volumes and function accurately, offer new opportunities for clinical use. Shorter acquisition times than the currently available methods for acquiring all imaging planes needed may also improve maternal comfort and cooperation, which is critical for achieving reliable fetal imaging in clinical practice.

## Funding

This work was supported by Region Skåne, Skånes universitetssjukhus, Hjärt–Lungfonden (20180510), Lund University Medical Faculty Foundation, Vetenskapsrådet (2019‐05506), and Siemens Healthineers.

## Conflicts of Interest

The authors declare no conflicts of interest.

## Supporting information


**Figure S1:** Representative images, computational performance, and VGC analysis for the evaluated reconstruction settings including 20 cardiac phases. This figure combines the results shown in Figures 3, 4, and 7.
**Figure S2:** Multiplanar reformatted CINE images in two cases: a healthy fetus (top row) and a fetus with double‐outlet right ventricle (bottom row). The left panels show SVD reconstructions and the middle panels show ROVir reconstructions, each reconstructed using 3 and 8 virtual channels and 8 and 16 cardiac phases. The right panels show the corresponding NUFFT non‐CINE reconstruction using root‐sum‐of‐squares coil combination.
**Figure S3:** All individual cases with corresponding cardiac regions of interest (cyan) and interference regions (red), alongside multiplanar reformatted CINE images comparing SVD and ROVir reconstructions. All reconstructions used 3 compressed channels and 16 cardiac phases. Red arrows indicate the right ventricle. Images are displayed after inter‐slice interpolation for visualization purposes.
**Figure S4:** Effect of varying the interference region in Subject 9. The cardiac region of interest (green) was kept fixed, while the interference region (red) was modified in position and size. Corresponding SVD (middle column) and ROVir (right column) root‐sum‐of‐square NUFFT reconstructions using 3 virtual channels are shown, demonstrating the robustness of ROVir to reasonable variations in interference region definition.
**Table S1:** Individual visual grading criteria and interobserver agreement.

**Video S1:** Video depicting longitudinal function in an oblique transversal view, comparing the SVD reconstruction (left) to the ROVir reconstruction (right) at a temporal resolution of 20 ms (Case2; fetal heart rate 148 bpm). Arrow pointing to the left ventricle.

**Video S2:** Video depicting ventricular function in the short‐axis view, comparing the SVD reconstruction (left) to the ROVir reconstruction (right) at a temporal resolution of 27 ms (Case1; fetal heart rate 113 bpm). Arrow pointing to the left ventricle.

## Data Availability

The data underlying this study cannot be shared publicly due to ethical and regulatory restrictions related to patient privacy. The code used for data processing and analysis is available from the corresponding author upon reasonable request.
