# Supplementary material for: Towards Faster Fetal 3D CINE Cardiac MRI Reconstruction Using Region‐Optimized Virtual Coils (ROVir)
Source: Magn Reson Med. 2026 Sep 22;97(1):159–68. doi: 10.1002/mrm.70590 (PMC13746063; doi:10.1002/mrm.70590)
Supplement: Supplementary file 1 [file MRM-97-159-s001.pdf]

Supporting information

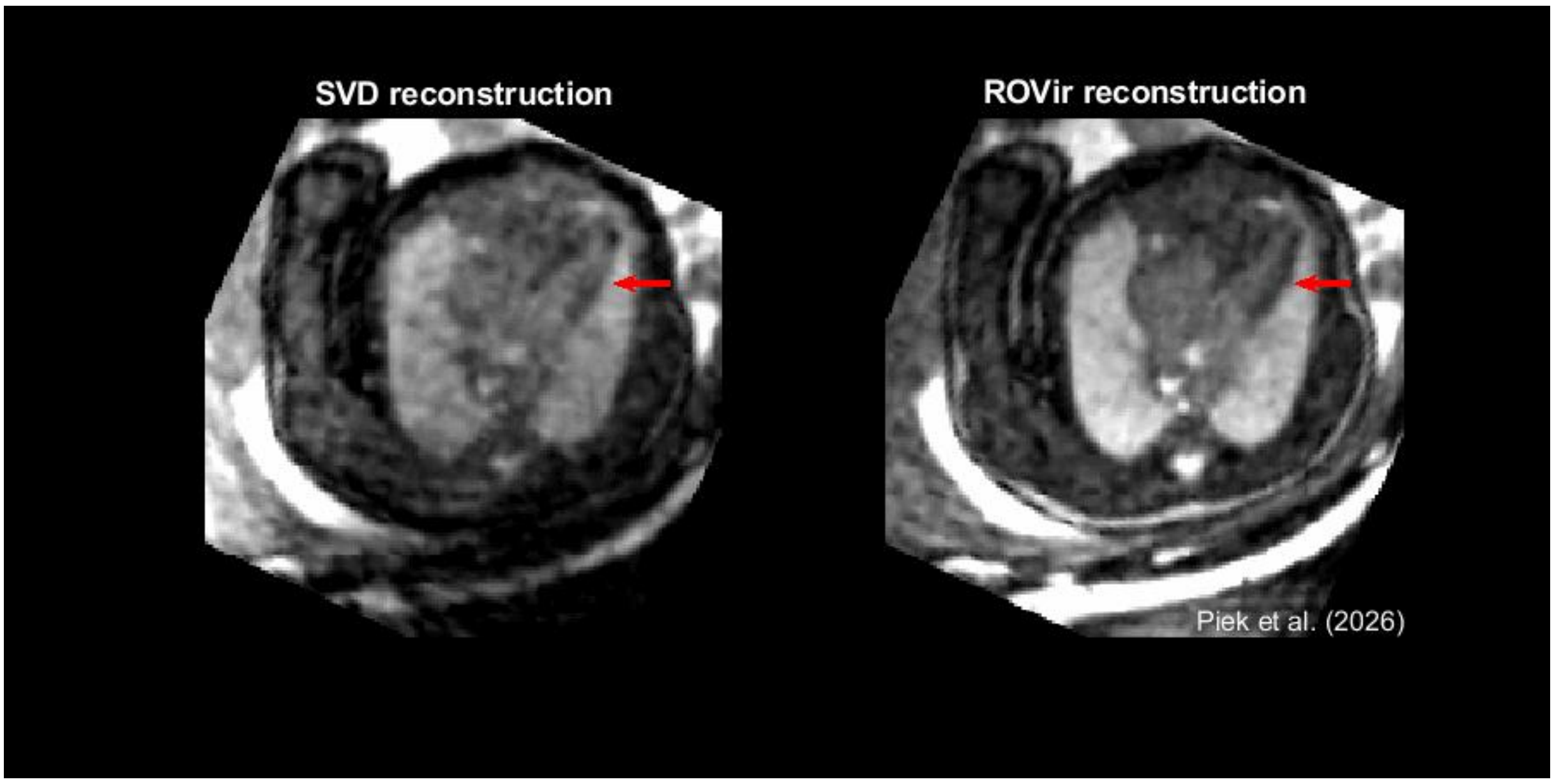

Video S1. Video depicting longitudinal function in an oblique transversal view, comparing the SVD reconstruction (left) to the ROVir reconstruction (right) at a temporal resolution of 20 ms (Case2; fetal heart rate 148 bpm). Arrow pointing to the left ventricle.

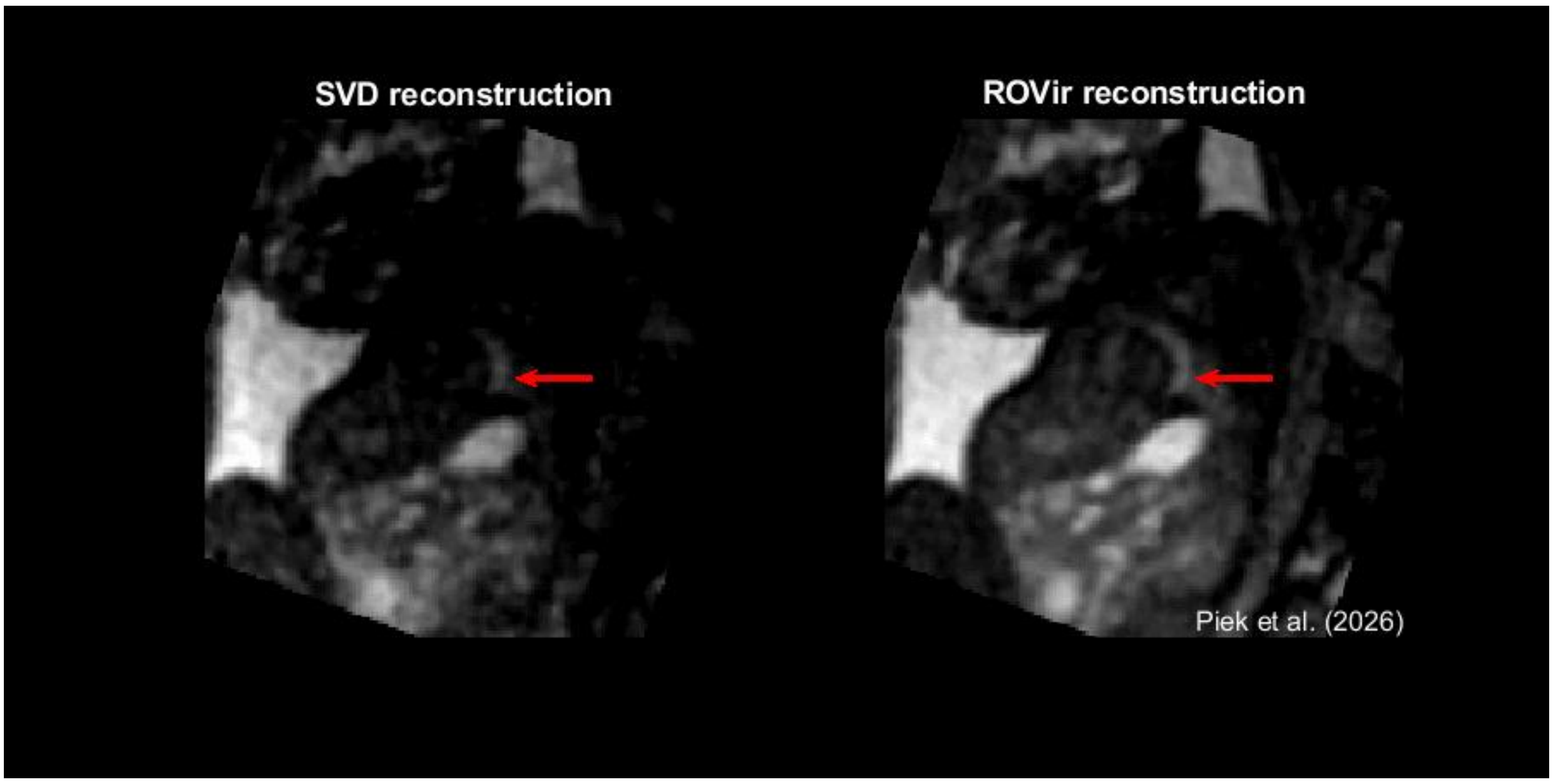

Video S2. Video depicting ventricular function in the short-axis view, comparing the SVD reconstruction (left) to the ROVir reconstruction (right) at a temporal resolution of 27 ms (Case1; fetal heart rate 113 bpm). Arrow pointing to the left ventricle.

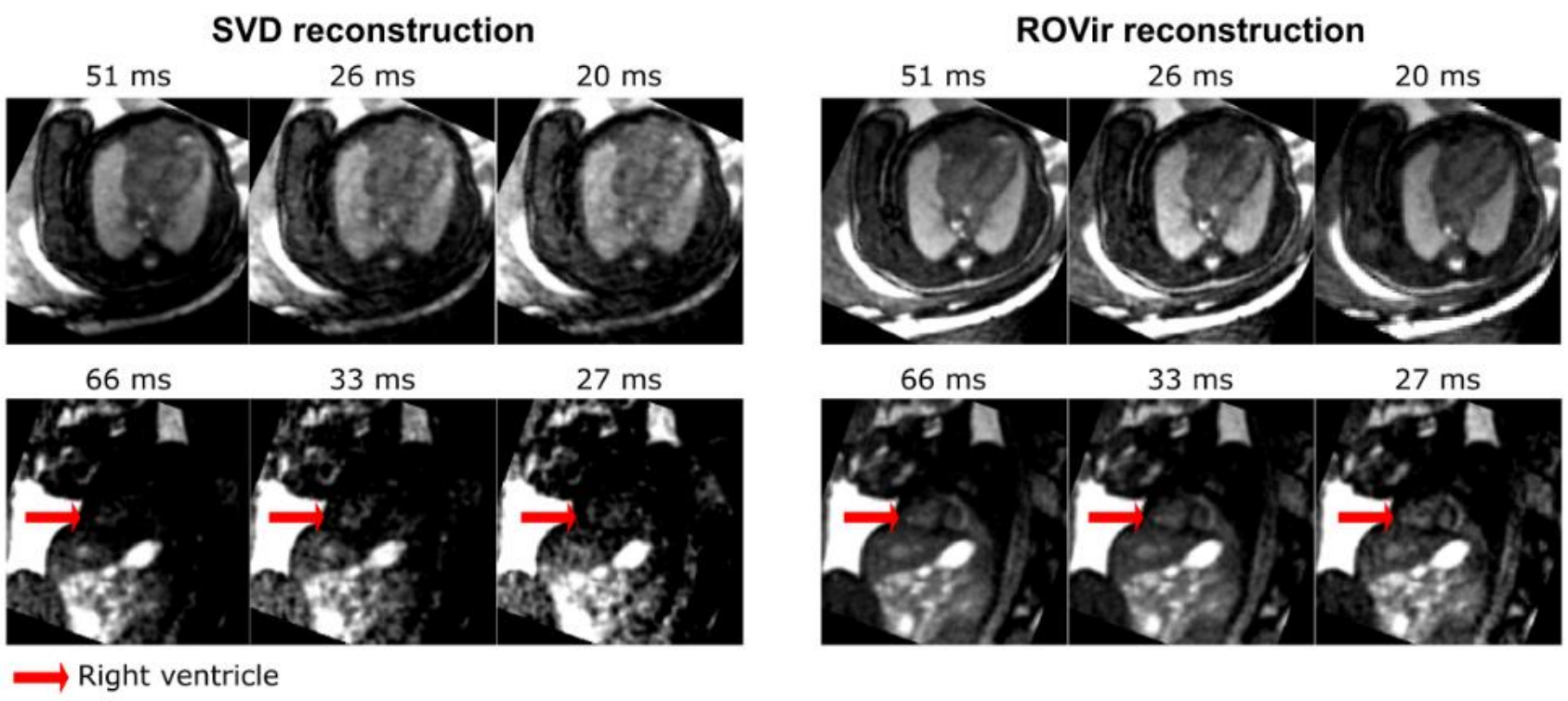
SVD reconstruction
ROVir reconstruction
51 ms
26 ms
20 ms
66 ms
33 ms
27 ms
Right ventricle

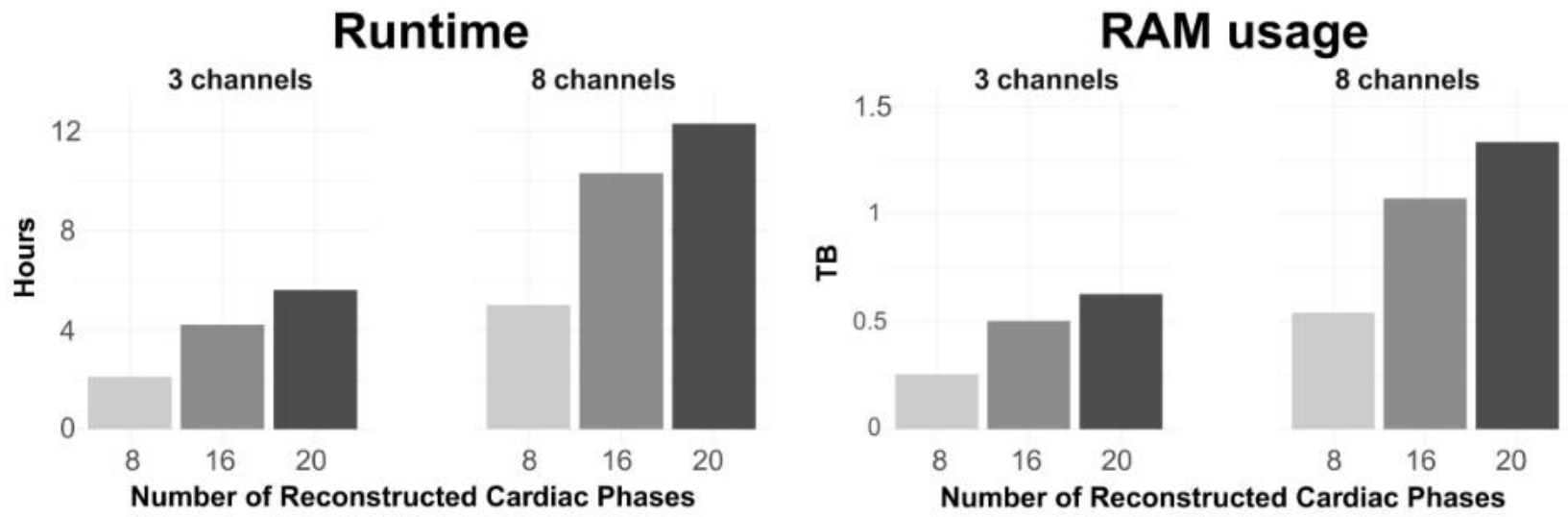
Runtime
RAM usage
3 channels
8 channels
Hours
TB
0
4
8
12
0.5
1
1.5
8
16
20
Number of Reconstructed Cardiac Phases

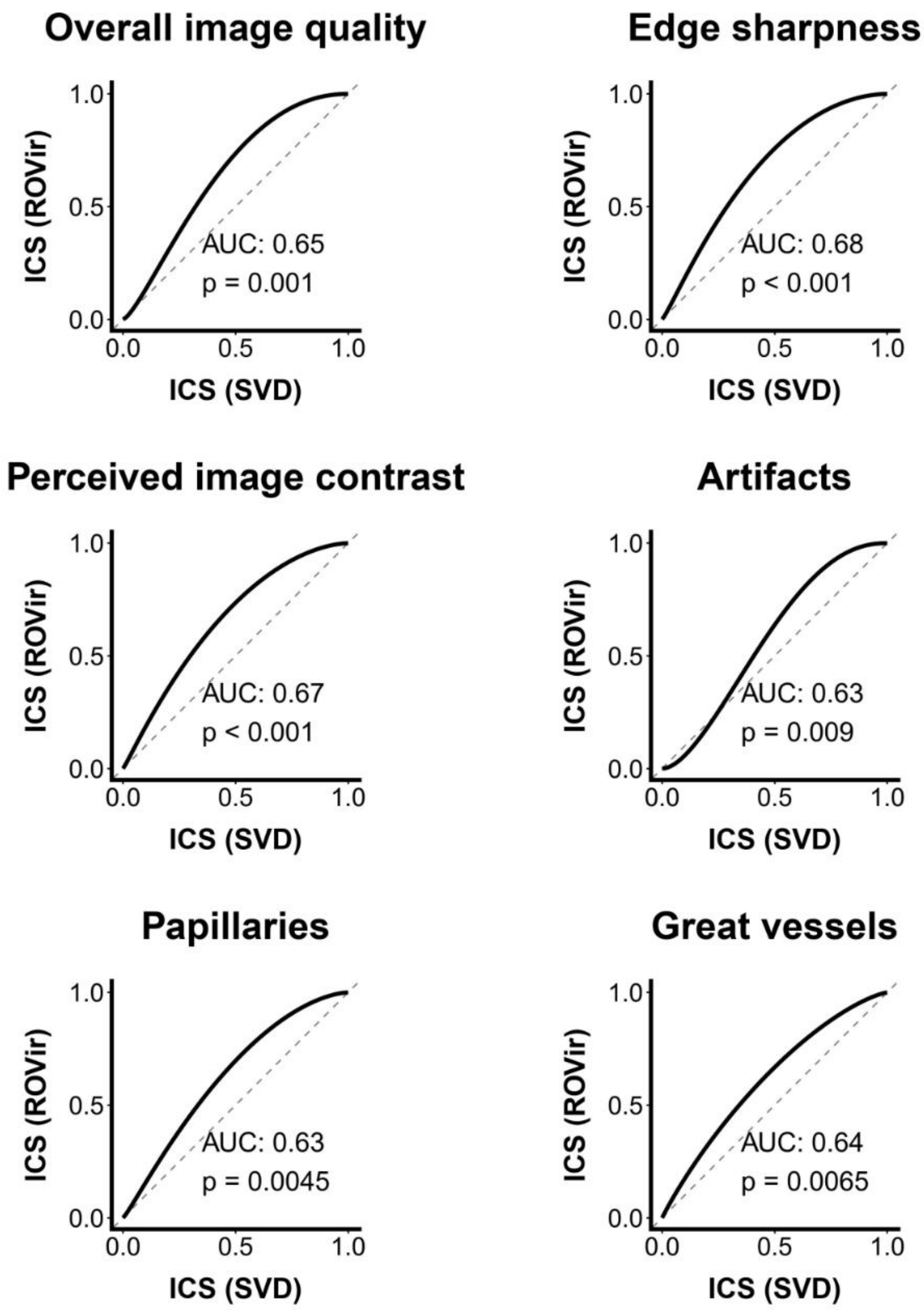
Overall image quality
AUC: 0.65
p = 0.001
Edge sharpness
AUC: 0.68
p < 0.001
Perceived image contrast
AUC: 0.67
p < 0.001
Artifacts
AUC: 0.63
p = 0.009
Papillaries
AUC: 0.63
p = 0.0045
Great vessels
AUC: 0.64
p = 0.0065
ICS (ROVir)
ICS (SVD)
0.0
0.5
1.0

Figure S1. Representative images, computational performance, and VGC analysis for the evaluated reconstruction settings including 20 cardiac phases. This Figure combines the results shown in Figures 3, 4, and 7.

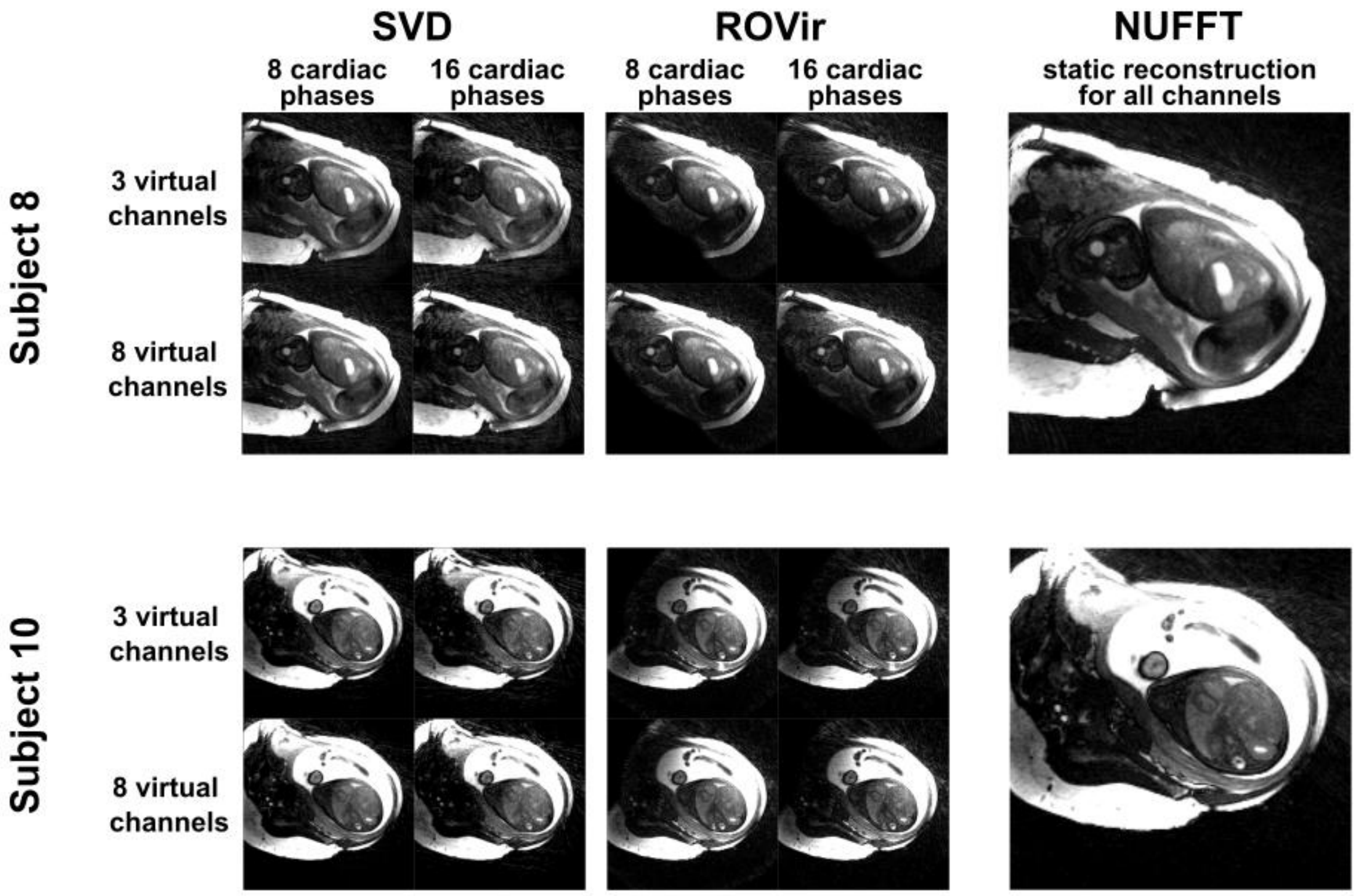

Figure S2. Multiplanar reformatted cine images in two cases: a healthy fetus (top row) and a fetus with double-outlet right ventricle (bottom row). The left panels show SVD reconstructions and the middle panels show ROVir reconstructions, each reconstructed using 3 and 8 virtual channels and 8 and 16 cardiac phases. The right panels show the corresponding NUFFT non-CINE reconstruction using root-sum-of-squares coil combination.

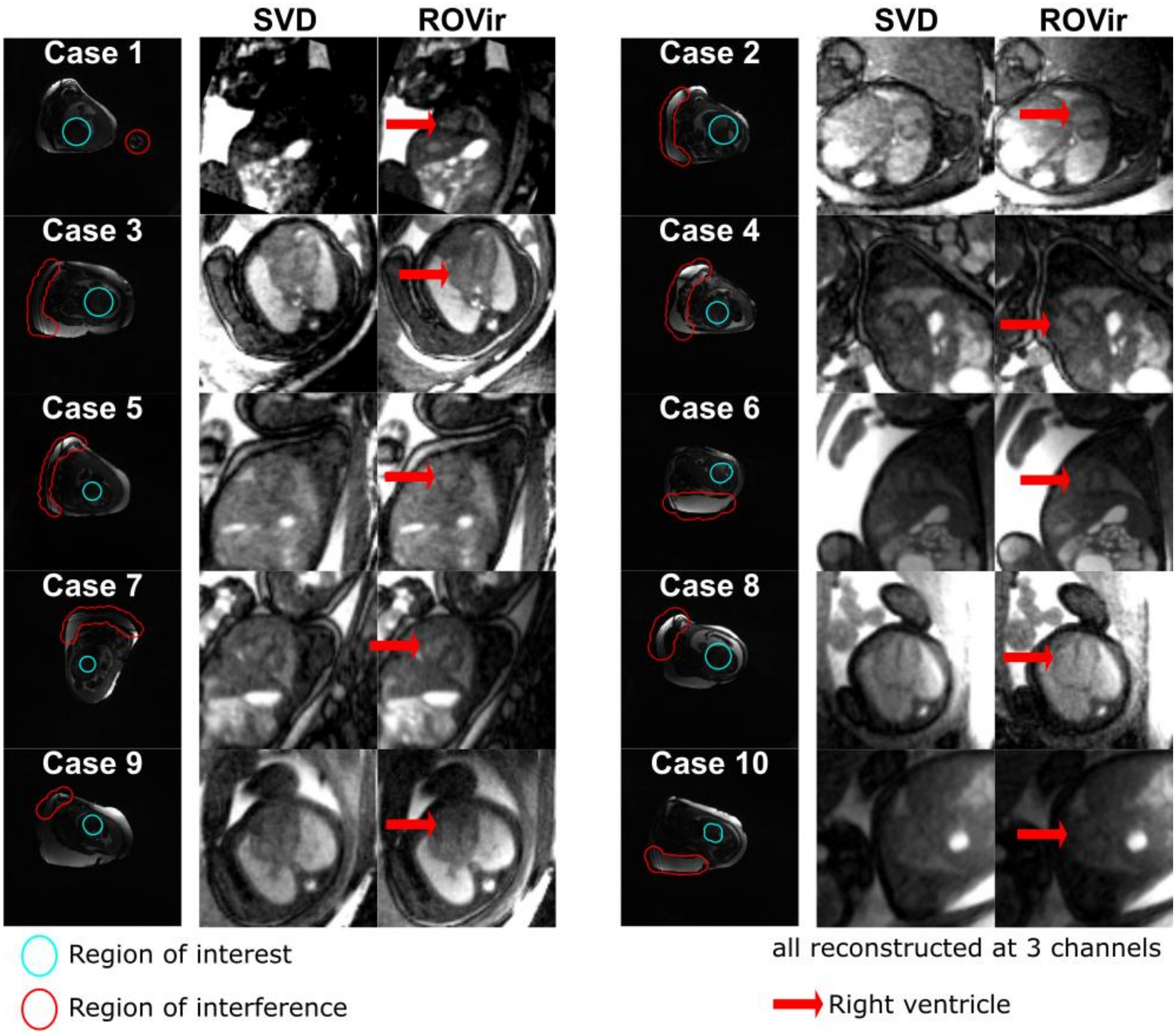

Figure S3. All individual cases with corresponding cardiac ROIs (cyan) and interference regions (red), alongside multiplanar reformatted cine images comparing SVD and ROVir reconstructions. All reconstructions used three compressed channels and 16 cardiac phases. Red arrows indicate the right ventricle. Images are displayed after inter-slice interpolation for visualization purposes.

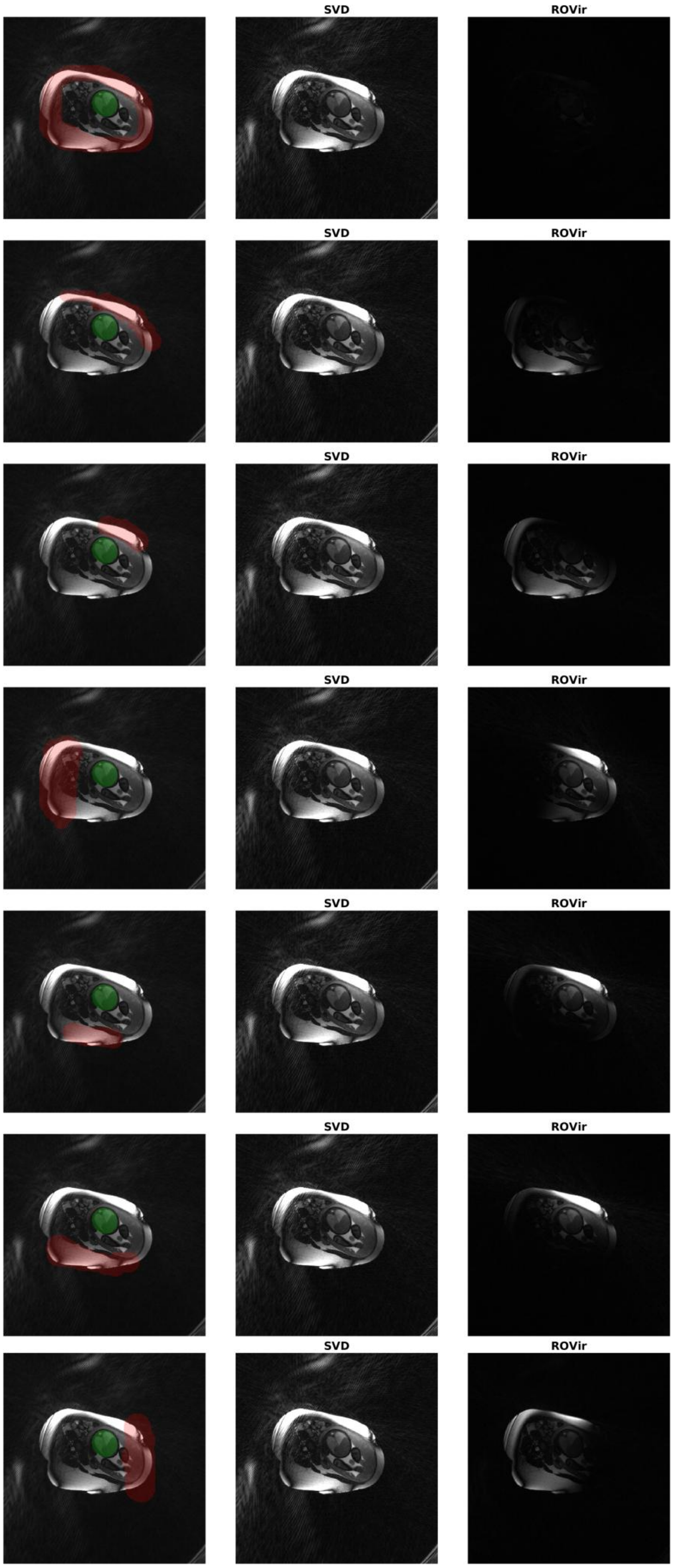
SVD
ROVir
SVD
ROVir
SVD
ROVir
SVD
ROVir
SVD
ROVir
SVD
ROVir
SVD
ROVir

Figure S4. Effect of varying the interference region in subject 9. The cardiac ROI (green) was kept fixed, while the interference region (red) was modified in position and size. Corresponding SVD (middle column) and ROVir (right column) root sum of square NUFFT reconstructions using three virtual channels are shown, demonstrating the robustness of ROVir to reasonable variations in interference region definition.

Table S1

Table S1. Individual visual grading criteria and interobserver agreement.

	Observer 1		Observer 2		
**Criterion**	**SVD**	**ROVir**	**SVD**	**ROVir**	**ICC(3,k)**
**Overall image quality**	4.7 (3.2 – 5.8)	5.1 (3.9 – 6.1)	2.5 (0.8 – 4.3)	2.7 (1.5 – 4.8)	0.63 (0.43 – 0.76)
**Edge sharpness**	2.5 (0.4 – 3.7)	3.0 (1.0 – 4.8)	1.8 (0.7 – 4.3)	2.4 (1.1 – 4.2)	0.70 (0.53 – 0.81)
**Perceived image contrast**	2.6 (0.4 – 4.4)	3.2 (1.0 – 5.0)	1.7 (0.7 – 4.0)	2.2 (1.1 – 4.7)	0.76 (0.62 – 0.84)
**Absence of artifacts**	6.3 (2.8 – 6.6)	6.3 (5.2 – 6.6)	1.9 (0.8 – 4.9)	2.3 (1.0 – 4.7)	0.47 (0.18 – 0.66)
**Papillaries**	2.2 (0.5 – 4.4)	2.8 (0.6 – 4.7)	2.8 (0.5 – 4.3)	3.2 (0.5 – 4.5)	0.67 (0.49 – 0.79)
**Great vessels**	3.6 (0.6 – 5.1)	4.1 (1.9 – 5.3)	1.6 (0.8 – 3.5)	1.9 (1.1 – 3.8)	0.61 (0.40 – 0.75)
**Extracardiac diagnostic image quality**	4.8 (3.5 – 5.4)	5.1 (4.4 – 5.7)	3.6 (2.5 – 4.8)	3.8 (2.7 – 4.8)	0.47 (0.17 – 0.66)

Scores are reported as median (2.5th–97.5th percentiles), for both observers. Interobserver agreement between the two observers was assessed using a two-way mixed-effects intraclass correlation coefficient (ICC) for consistency of average measurements. SVD = Singular Value Decomposition, ROVir = Region Optimized Virtual coils.
